# Supplementary material for: Towards unified AI-driven fracture mechanics: the extended deep energy method (XDEM)
Source: Nat Commun. 2026 Aug 15;17:8492. doi: 10.1038/s41467-026-76748-1 (PMC13484471; doi:10.1038/s41467-026-76748-1)
Supplement: Supplementary file 3 [file 41467_2026_76748_MOESM3_ESM.zip › pic/Results/Crack_propagation/Straight/3D_dis_force/Tension_3D/1dPhi_16.pdf]

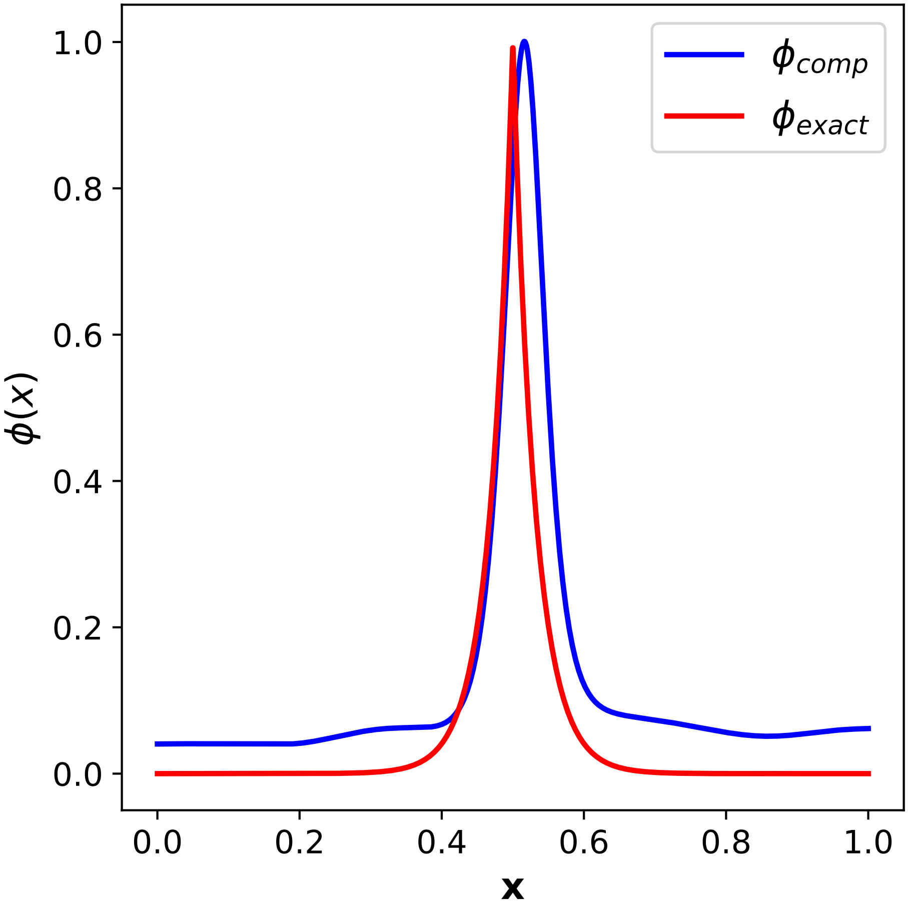

$\phi_{comp}$
$\phi_{exact}$
$\phi(x)$
x
0.0
0.2
0.4
0.6
0.8
1.0
